# Motivational sensitivity of outcome-response priming: Experimental research and theoretical models

**DOI:** 10.3758/s13423-018-1449-2

**Published:** 2018-02-21

**Authors:** Poppy Watson, Reinout W. Wiers, Bernhard Hommel, Sanne de Wit

**Affiliations:** 1 ADAPT lab, Department of Developmental Psychology, University of Amsterdam, Amsterdam, Netherlands; 2 Amsterdam Brain and Cognition, University of Amsterdam, Amsterdam, Netherlands; 3 Habit Lab, Department of Clinical Psychology, University of Amsterdam, Nieuwe Achtergracht 129-B, 1018 WS Amsterdam, Netherlands; 4 Cognitive Psychology Unit, Leiden University, Leiden, Netherlands; 5 Leiden Institute for Brain and Cognition, Leiden, Netherlands

**Keywords:** Ideomotor theory, Response priming, Motivation, Goal-directed action, Pavlovian-to-instrumental transfer

## Abstract

Outcome-response (O-R) priming is at the core of various associative theories of human intentional action. This is a simple and parsimonious mechanism by which activation of outcome representations (e.g. thinking about the light coming on) leads to activation of the associated motor patterns required to achieve it (e.g. pushing the light switch). In the current manuscript, we review the evidence for such O-R associative links demonstrated by converging (yet until now, separate) strands of research. While there is a wealth of evidence that both the perceptual and motivational properties of an outcome can be encoded in the O-R association and mediate O-R priming, we critically examine the integration of these mechanisms and the conditions under which motivational factors constrain the sensory O-R priming effect. We discuss the clinical relevance of this O-R priming mechanism, whether it can satisfactorily account for human goal-directed behaviour, and the implications for theories of human action control.

How are intentions translated into actions? Knowledge of the relationship between actions and the outcomes that they produce is an essential prerequisite for goal-directed behaviour. If I wish to turn the light on, then prior experience tells me that this can be achieved by pushing the light switch (and not, for example, a button on the TV remote control). Many different associative theories are based upon the central idea that in the course of exploration and learning, associative links between responses (R) and outcome (O) representations are formed (Asratyan, 1974; Gormezano & Tait, 1976; Hommel, Müsseler, Aschersleben, & Prinz, 2001; James, 1890). As a consequence, activation of the outcome representation (thinking about the light coming on) leads to activation of the associated motor patterns required to achieve it (pushing the light switch). Evidence for such O-R associative links comes from multiple converging strands of research showing that presentation (or anticipation) of outcomes activates associated motor responses and that preparing motor responses activates anticipation of outcomes. But how and under what circumstances do motivational factors constrain such effects? In the current manuscript, we review O-R priming effects, focusing on the integration of sensory and motivational aspects of action control.

## Theories of action control

Various models of human behaviour contain an O-R mechanism that either partly or fully drives action control. Investigations into O-R priming effects have been conducted in the fields of both human psychology and animal learning, although these two research traditions have remained relatively separate and have maintained a separate emphasis of investigation. Ideomotor theorists (e.g. Hommel, 2009; Hommel et al., 2001; James, 1890; Lotze, 1852) have tended to focus on how perceptual and sensory outcomes (or ‘action effects’) are translated into appropriate motor sequences in humans and the factors that affect the frequency, speed, and efficiency of this process. By contrast, researchers from the field of animal associative learning have mostly used motivationally relevant outcomes (such as food; e.g. Asratyan, 1974; Gormezano & Tait, 1976; Pavlov, 1927) and directly investigated the conditions under which actions are not only driven by knowledge of (perceptual) O-R relationships but also modulated by changes in the current motivational significance of those outcomes (Adams & Dickinson, 1981). Based on this work (the findings of which are discussed in more detail below; see section Modulation of O-R Priming by Changes in Outcome Value) some theories of action control, such as recent formulations of the associative-cybernetic model (S. de Wit & Dickinson, 2009), include an O→R mechanism as one path to action but supplement this with a forward R→O pathway to fully capture goal-directed action control.

In recent years, many human studies have been conducted with the aim of shedding light on the role of sensory and motivational outcomes in O-R priming. In the remainder of this manuscript, we will review research investigating the O-R mechanism, including studies that have utilized ideomotor O-R priming paradigms and paradigms derived from research into animal learning. We will then assess the degree to which this O-R priming mechanism is modulated by motivational factors and discuss whether a simple O-R model can be a sufficient account of intentional human behaviour.

It should be noted that there are differing views on how the associative links between responses and the outcomes they produce are formed. The bidirectional hypothesis assumes that bidirectional R-O associations are formed during training as a consequence of the causal relationship between the instrumental response and the outcome, allowing for later ‘backwards’ response priming in the O-R direction (Elsner & Hommel, 2004; Pavlov, 1932; Rescorla, 1992). Others have argued that contextual stimuli generate expectancy of the outcome (“O”) that precedes the response, leading to the formation of O-R associations (where the associatively retrieved outcome representation effectively functions as an antecedent stimulus; Trapold & Overmier, 1972). O-R links can also be generated in blocked designs where single instrumental response contingencies are trained separately (i.e. R1-O1-R1-O1 in one block and R2-O2-R2-O2 in another block, as is common in animal studies; Ostlund & Balleine, 2007). These blocked designs ensure that the outcome presentation of one trial precedes execution of the response, and can thus function as a discriminative cue (i.e. O1 primes R1 and O2 primes R2). Evidence for different types of O-R associations has been reported (Alarcón, Bonardi, & Delamater, 2017; Gilroy, Everett, & Delamater, 2014; Ostlund & Balleine, 2007; Rescorla, 1992). Distinguishing between these various accounts is beyond the scope of the current manuscript, although the implications for understanding the role of motivation are discussed in more detail below (see section Implications for Theories of Action Control).

## Outcome anticipation and O-R priming

In this section, we review studies that have investigated outcome anticipation and the sensory and affective components of outcome representations. We also review evidence for the O-R priming mechanism from various strands of research utilizing instrumental discrimination paradigms and response-priming tasks in which outcomes are presented either directly to participants or are signalled indirectly (via Pavlovian cues).

### Representation of sensory and affective outcomes

The consequences of our outcomes are subjectively perceived to occur earlier in time (closer to the response) than responses that were carried out by others or are unexpected—an effect known as intentional binding (Moore & Obhi, 2012). Furthermore, the sensory properties of produced outcomes are attenuated, both subjectively and in terms of their cortical response (Desantis, Roussel, & Waszak, 2014). These findings are often attributed as evidence for sensory O-R binding that occurs when we anticipate outcomes. Some researchers have used neuroimaging and electrophysiological techniques to more directly demonstrate anticipation of sensory outcomes (Band, van Steenbergen, Ridderinkhof, Falkenstein, & Hommel, 2009; Kühn & Brass, 2010; Kühn, Keizer, Rombouts, & Hommel, 2010; Pfister, Melcher, Kiesel, Dechent, & Gruber, 2014; Vincent, Hsu, & Waszak, 2016; Waszak & Herwig, 2007; Zwosta, Ruge, & Wolfensteller, 2015). In the study of Kühn et al. (2010), for example, participants were asked to prepare either hand or facial actions, during which anticipatory activations in the relevant perceptual areas (extrastriate body area and fusiform face area, respectively) were observed. In an attempt to compare sensory and affective outcome representations, Vincent and colleagues used EEG and investigated the prediction error signal generated by unexpected outcomes (Vincent et al., 2016). Participants pushed four response keys that consistently yielded the same picture of a face (either an adult’s or child’s face with either a positive or negative expression). However, occasionally a key press would yield an unexpected picture—these could differ across category (e.g. a child’s face would be presented instead of an adult’s) or could differ across valence (e.g. a positive child’s face would be presented instead of a negative child’s face) or could differ across both dimensions. The authors demonstrated that all unexpected outcomes, whether differing across category, valence, or both dimensions, generated a similar prediction error signal leading them to conclude that the affective and sensory aspects of an outcome are represented together.

### Instrumental discrimination studies

The role of outcome anticipation in action selection has been investigated with a variety of instrumental discrimination paradigms, in both animals and humans, in which anticipated outcomes interfere with, or facilitate, ongoing actions. De Wit and colleagues, for example, showed that participants learned to perform biconditional instrumental S:R→O discriminations at a slower rate when the discriminative stimulus (a fruit image) preceding one response was the same as the outcome (a fruit image) following a different response (S. de Wit, Corlett, Aitken, Dickinson, & Fletcher, 2009; S. de Wit, Niry, Wariyar, Aitken, & Dickinson, 2007; S. de Wit, van de Vijver, & Ridderinkhof, 2014). For example, in the easy, congruent discrimination, a picture of an orange signalled that pressing right would be rewarded with an orange. In contrast, in the incongruent discrimination, a picture of a pear signalled that pressing right led to an apple, while on other trials an apple stimulus signalled that pressing left was rewarded with a pear. This interference comes about because the response signalled by the discriminative stimulus (S-R) conflicts with the response triggered by the outcome anticipation (O-R priming).

Similarly, the ‘differential outcomes effect’ refers to the phenomenon that discriminative learning of multiple instrumental stimulus-response-outcome (S-R-O) relationships is superior when multiple unique outcomes are employed (e.g. S1:R1-O1; S2:R2-O2) compared with when the outcome is the same across the different S-R-O relationships (e.g. S:R1-O1; S2:R2-O1; Mok & Overmier, 2007; Trapold, 1970; for review, see Urcuioli, 2005). It is argued that in the latter condition, anticipation of the instrumental outcome activates both associated responses via O-R associations, regardless of which response is signalled to be correct by the discriminative stimulus. The ‘differential outcomes effect’ provides support, therefore, for the O-R mechanism. This effect can be observed not only with rewarding outcomes (Trapold, 1970) but also with purely sensory outcomes, (e.g., Fedorchak & Bolles, 1986).

In an example of response *facilitation* by outcome anticipation, a number of studies have shown that responses followed by perceptually congruent outcomes are executed faster (Gaschler & Nattkemper, 2012; Pfister, Kiesel, & Hoffmann, 2011; Pfister, Kiesel, & Melcher, 2010). This perceptual congruency effect was demonstrated by Pfister et al. (2010), who showed that, for example, right responses were carried out faster when the associated outcome was presented on the right side of the screen relative to when the outcome was presented on the left (as is observed with stimulus-response spatial congruency in the classic Simon effect; Simon & Berbaum, 1990; Simon & Rudell, 1967). It is clear, however, that particular task setups can reduce the impact of outcome anticipation on ongoing response selection. The use of very simple, explicitly instructed, stimulus-response mappings seem to eradicate the facilitatory effects of perceptually congruent responses and outcomes (Gozli, Huffman, & Pratt, 2016; Herwig, Prinz, & Waszak, 2007; Herwig & Waszak, 2009; Pfister et al., 2011; Pfister et al., 2010; Zwosta, Ruge, & Wolfensteller, 2013).

### Direct O-R priming

Direct presentation of outcomes can also trigger responses that previously led to them. In a line of research that originates in animal studies, researchers studying reinstatement have utilized direct O-R priming using food (and drug) rewards. For example, in rats, consumption of a small amount of food has been shown to reinstate a previously extinguished response that used to yield that reward (Ostlund & Balleine, 2007; for review, see H. de Wit, 1996). Likewise in humans, it has been demonstrated that presentation of the rewarding outcome (e.g. picture of a food or drug outcome) on a computer screen can also prime associated responses (Hogarth, 2012; Hogarth & Chase, 2011; Watson, Wiers, Hommel, Ridderinkhof, & de Wit, 2016). For example, Hogarth and Chase (2011) showed that presenting pictures of chocolate or cigarettes on-screen selectively increased responding on a key that previously yielded the depicted rewards.

**Fig. 1:**
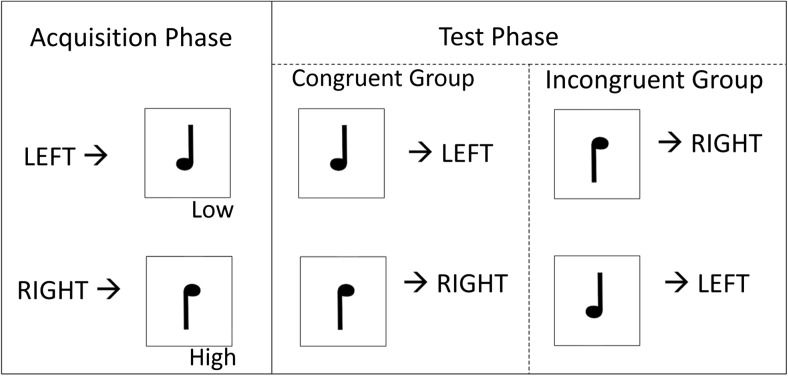
Classic two-stage ideomotor paradigm. During the test phase, the outcomes now function as discriminative stimuli, and participants in the incongruent group are instructed to make the opposite response

Ideomotor theorists developed an alternative way to assess O-R priming with the classic two-stage ideomotor paradigm in which novel S-R instructions interfere with previously learned O-R associations (Elsner & Hommel, 2001). During the training phase, participants learned the relationships between responses and outcomes. For example, a right key press was always followed by a high-pitched tone, and a left key press was followed by a low-pitched tone (see Fig. 1 for schematic). In the test phase, the two tones were presented as discriminative stimuli, and participants were either instructed to make the same response as during training (congruent mapping group; e.g. a high tone should be followed by a right key press) or were asked to make the opposite response to that which was learned during training (incongruent mapping group; e.g. the high tone should be followed by a left key press). Elsner and Hommel (2001) showed that participants in the incongruent group were slower to respond than those in the congruent group, suggesting that presentation of the tone outcomes automatically elicited the associated behavioural response, which then interfered with selection of the correct (incongruent) response. Using similar designs, this effect has been replicated hundreds of times (for review, see Shin, Proctor, & Capaldi, 2010), although the two-stage paradigm does appear to be difficult to scale up to more complex situations (Watson, van Steenbergen, de Wit, Wiers, & Hommel, 2015). There is also evidence to suggest that such response priming can occur even when the outcomes are not consciously perceived during the test phase (Kunde, 2004). The strength of the two-stage paradigm is that subtle RT effects as the result of O-R priming can be detected independently of explicit intentions to perform specific responses. In other words, O-R priming effects are less likely to be the result of explicit strategies (e.g. upon hearing the high-pitched tone, “the experimenter probably wishes me to press the key that previously led to this outcome”). However, studies using the two-stage paradigm to study direct O-R priming in humans have used purely sensory (perceptual) outcomes, such as shapes and tones, that have limited motivational significance.

A possible O-R priming effect has also been demonstrated by Aarts and Dijksterhuis (2000a, 2000b) using their ‘goal-priming’ paradigm. In a typical study of this series, travel destinations were used that, during a pilot study, had already been identified as destinations where nearly everybody either cycled or took the train. During the task, these destinations were presented on the screen as discriminate stimuli, and participants had to respond (verbally) with either a typical (i.e. bike/train) or atypical mode of travel. Participants in the atypical condition made more errors, suggesting that the destination outcome triggered a ‘typical mode of travel’ response via an O-R priming mechanism. While this paradigm arguably has strong ecological validity, it is difficult to assess the precise underlying mechanisms driving such an effect.

### Pavlovian-to-instrumental transfer (PIT)

**Fig. 2:**
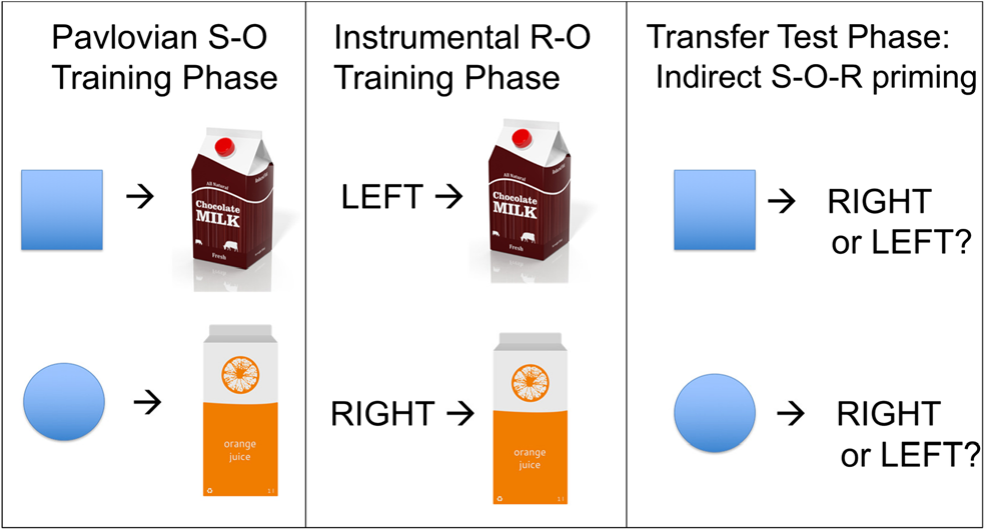
Classic Pavlovian-to-instrumental transfer paradigm. The integration of separately learned S-O and O-R associations are examined in a test phase in which the Pavlovian stimuli are presented and response choice measured. Indirect O-R priming (PIT) occurs when anticipation of the chocolate milk (generated by the square stimulus) causes participants to push more on the left (chocolate-milk-yielding) key

Seeing someone enjoy a large slice of chocolate cake can trigger a trip to the bakery, but even merely being reminded of chocolate cakes by environmental cues is sufficient to lead to the bakery-visiting response. This *indirect* priming of instrumental responses by environmental cues can be demonstrated using the outcome-specific PIT task, which has been extensively used in animal research (for review, see Cartoni, Balleine, & Baldassarre, 2016; Holmes, Marchand, & Coutureau, 2010; Rescorla & Solomon, 1967) but more recently also in human studies. To illustrate, participants in the experiment of Bray, Rangel, Shimojo, Balleine, and O’Doherty (2008) first underwent Pavlovian S-O training and learned the relationships between simple geometric shapes and drink outcomes (e.g. a square predicted delivery of chocolate milk and a circle predicted delivery of orange juice; see Fig. 2). In a separate instrumental R-O training phase, they then learned that a left key press yielded chocolate milk and a right key press yielded orange juice. In the transfer test phase (conducted in extinction), participants were free to respond on either response key while occasionally the Pavlovian cues were presented. The classic outcome-specific PIT effect was observed such that the square (previously associated with the chocolate milk) caused participants to respond more on the left key, while the circle (associated with orange juice) biased responding towards the right key. As the Pavlovian stimuli had never been directly paired with either response, it is argued that the Pavlovian stimuli elicited anticipation of the outcome, which then activated the associated motor response (indirect S-O-R priming).

Other human PIT studies have employed similar designs with different types of motivationally relevant outcomes, such as food rewards (Bray et al., 2008; Eder & Dignath, 2016b; Morris, Quail, Griffiths, Green, & Balleine, 2015; Prévost, Liljeholm, Tyszka, & O’Doherty, 2012; Quail, Morris, & Balleine, 2016; Watson, Wiers, Hommel, & de Wit, 2014; Watson et al., 2016); cigarette, alcohol, and monetary rewards (Allman, DeLeon, Cataldo, Holland, & Johnson, 2010; Eder & Dignath, 2016a; Hogarth, Dickinson, Wright, Kouvaraki, & Duka, 2007; Jeffs & Duka, 2017; Martinovic et al., 2014); but also more abstract rewards (e.g. points: Nadler, Delgado, & Delamater, 2011; Paredes-Olay, Abad, Gámez, & Rosas, 2002). The PIT effect appears, therefore, to be relevant for understanding behaviours generated towards procurement of appetitive outcomes in our environment.

Of course, much of our instrumental behaviour is also directed towards the prevention of aversive outcomes occurring. To this end, avoidance PIT paradigms have also been developed—where Pavlovian stimuli signal an aversive outcome—causing participants to make a response that during instrumental training prevented that outcome from occurring (Campese, McCue, Lázaro-Muñoz, LeDoux, & Cain, 2013; Garofalo & Robbins, 2017; Lewis, Niznikiewicz, Delamater, & Delgado, 2013). Relatedly, a number of studies have also investigated conditioned inhibition in PIT (Alarcón & Bonardi, 2016; Laurent & Balleine, 2015; Quail, Laurent, & Balleine, 2017). During Pavlovian training, a particular CS is always reinforced, unless it is presented alongside the conditioned inhibitor—a CS whose presence signals the *absence* of that particular reward. In line with the idea that the conditioned inhibitor suppresses the outcome representation, O-R priming is reduced in the presence of the conditioned inhibitor (Alarcón & Bonardi, 2016; Quail et al., 2017), and in some situations, responding for the alternative reward is boosted (Laurent & Balleine, 2015).

We should note that a related group of studies have used a simpler version of the PIT paradigm, in which only a single response was trained (e.g. S1-O followed by R1-O) to show the motivating (and inhibitory) effects of Pavlovian cues on ongoing appetitive (and avoidance) responses towards either monetary or chocolate rewards (in humans; Colagiuri & Lovibond, 2015; Garbusow et al., 2015; Garofalo & di Pellegrino, 2015; Guitart-Masip et al., 2011; Lovibond & Colagiuri, 2013; Talmi, Seymour, Dayan, & Dolan, 2008). However, because these studies only included one instrumental response, it is unclear whether the facilitatory effect observed is a specific O-R priming effect or whether the Pavlovian cues boosted the motor system generally, and thereby increased overall response vigour (an effect known as ‘general PIT’; Chiu, Cools, & Aron, 2014; Corbit & Balleine, 2005; Corbit, Janak, & Balleine, 2007; Holland, 2004). We know that this general effect can occur from elegant studies that disentangle specific and general PIT effects. For example, Corbit and Balleine (2005) showed within a single paradigm that Pavlovian stimuli for instrumental outcomes (CS1-O1 and CS2-O2) would specifically enhance performance of responses that previously led to those outcomes (R1-O1 and R2-O2), while a CS for a third noninstrumental outcome led to increased performance of both (R1 and R2) responses relative to baseline. The general motivating effect of Pavlovian cues on ongoing response behaviour is reduced if the general outcome is not currently desired (Corbit et al., 2007; Watson et al., 2014).

## Motivational modulation of O-R priming

As has been outlined in preceding sections, a wealth of evidence shows that O-R priming is a simple mechanism that explains how anticipation of outcomes can lead to the selection of the appropriate responses that will result in that outcome (or prevention of an aversive outcome). There is also evidence that both the perceptual and motivational properties of an outcome can be encoded in the outcome representation. A more complex question, however, is whether the motivational significance of outcomes constrains whether or not the associated action is carried out. If, as evidence suggests, outcome presentation (or mere anticipation) can trigger responses associated with similar perceptual and affective outcomes, it begs the question of why we are not automatons, stuck in endless action loops whereby outcomes in the environment constantly trigger actions, triggering outcomes, triggering actions, and so forth (Konorski, 1967; Pezzulo, Baldassarre, Butz, Castelfranchi, & Hoffmann, 2007). Clearly, our behaviour needs to be constrained in a specific manner by motivational factors, namely, “is this outcome worth pursuing *at this moment in time*”? Being reminded of chocolate cakes may activate the associated response representation (head to the bakery), but to what degree is activation or its impact on action control mediated by the degree to which the chocolate cake is currently desired? In the following sections, we first review studies that have shown that outcome value can mediate the O-R priming effect and then assess the evidence for modulation by the current desirability of outcomes.

### Contrasting O-R priming by high-value and low-value outcomes

Using the classic two-stage ideomotor paradigm an interesting set of studies have contrasted positive and negative outcomes and subsequent priming of actions that previously led to a different, yet affectively similar, outcome (Beckers, De Houwer, & Eelen, 2002; Eder, Rothermund, De Houwer, & Hommel, 2014; Lavender & Hommel, 2007). Participants in the study of Beckers et al. (2002) first underwent R-O training, learning that one response was followed by an electric shock and another response was not. In the test phase, participants saw words (either positive or negative) and were instructed to make one response for verbs and the other for nouns (using the same two response keys as during the training phase). An affective congruency effect was observed such that the response associated with the electric shock was carried out faster for negatively valenced words while the other response (associated with the absence of shock) was carried out faster for positive words. Similar results were found by Eder et al. (2014) using positive and negatively valenced pictures during the training phase rather than electric shocks. Related studies used compound stimuli during a test phase to examine whether a CS predictive of an aversive shock would bias participants to carry out that action (Claes, Crombez, Franssen, & Vlaeyen, 2016; Claes, Vlaeyen, & Crombez, 2016). In one of these studies, for example, participants were presented with two discriminative stimuli signalling that one response would be punished with an electric shock and the other reinforced with a lottery ticket. Each of these discriminative stimuli was then combined with a coloured shape that during a Pavlovian training phase had signalled either the reward or the aversive shock. In contrast to the aforementioned studies, the authors did not find any evidence for increased responding for the aversive shock outcome in the presence of the electric shock CS (Claes, Crombez, et al., 2016; Claes, Vlaeyen, et al., 2016). However, the tests in these studies were not performed in extinction (the shock outcome was delivered if participants made the shock response), meaning that participants were able to continually adjust their behaviour based on the aversive feedback. In addition the explicit choice between the two outcomes (offered by the two discriminative stimuli) might have reduced any O-R priming effects (a point we return to later). This is, nonetheless, an intriguing paradigm and could be used to explore further the conditions under which O-R priming is mediated by the aversive properties of an outcome. The existing evidence that a response that previously led to an aversive outcome can be primed more readily in some situations (Beckers et al., 2002; Eder et al., 2014) is counterintuitive when we consider the role of this mechanism in goal-directed behaviour, a point that we will return to in a later section.

In another study using food outcomes, Watson et al. (2016) examined both direct O-R priming (with pictures of food outcomes that had been associated during the training phase with particular responses) and indirect S-O-R priming (using Pavlovian stimuli that had previously been associated with those food pictures, but never with a response). In an instrumental learning phase, discriminative stimuli signalled whether a left or right key was the correct response and whether it would be rewarded with a picture of a palatable, high-calorie outcome or with a relatively bland, low-calorie food picture. Each response key was assigned to one high-calorie and one low-calorie outcome (e.g. S1: R1→potato chips; S2: R2→ chocolate; S3: R1→lettuce; S4: R2→courgette). This design ensured that there was no baseline response preference based on the calorie content of the food outcomes, thereby allowing for independent assessment of the effect of outcome value on O-R priming. To this end, during the test phase, participants saw the food pictures (or Pavlovian stimuli previously associated with the food pictures) and were asked to spontaneously select a key as quickly as possible, every time that a picture appeared. Even though participants did not sample the food during the task (only beforehand in a taste test), results showed that the palatable, high-calorie food pictures (or Pavlovian stimuli previously associated with these) more frequently primed the relevant instrumental response, relative to the low-calorie food outcomes. A similar but more complex design was used by Muhle-Karbe and Krebs (2012) to show that when used as task-irrelevant primes, high-value outcomes interfere more with explicit task instructions. Using a two-stage design, responses were first associated with coloured squares (where the colour indicated the reward value). During the second phase, participants were explicitly told that no rewards would be given. A new set of discriminative stimuli signalled the correct response to make. The coloured squares (outcomes from Phase 1) were then presented as task-irrelevant primes (just before the discriminative stimulus) and could be either congruent or incongruent in respect to the previous response mapping. The authors found that incongruent responses were carried out slower on trials that were primed by the high-reward colour, suggesting that the presentation of the outcome in Phase 2 triggered the previously learned response (via an O-R mechanism) and that this priming effect was more difficult to overcome in the high-value condition. In addition, Muhle-Karbe and Krebs (2012) found that the degree to which high-reward primes interfered with performance on incongruent trials was related to a self-report measure of reward sensitivity. Taken together, these two studies suggest that the O-R priming mechanism is sensitive to outcome value and that O-R priming is more pronounced in the context of high-reward outcomes.

Another set of studies have attempted to investigate O-R priming in more ecologically valid experiments, for example, using task setups where multiple outcomes of various reward value are in view rather than only one outcome (or Pavlovian CS) being visible on each trial. These studies suggest that the affective properties of outcomes can have subtle yet measurable effects on ongoing responses directed towards an outcome in another location, by biasing the trajectories of movements in the direction of the alternative (not to be approached) outcome (Dignath, Pfister, Eder, Kiesel, & Kunde, 2014; Herwig & Horstmann, 2011; Hommel, Lippelt, Gurbuz, & Pfister, 2016; Pfister, Janczyk, Wirth, Dignath, & Kunde, 2014). This work, in which O-R priming is investigated in a richer environment, offers an interesting avenue for future research—although it would be interesting to examine situations when interference from alternative outcomes is definitely mediated by learned O-R associations (and cannot simply be the result of interference by a Pavlovian approach response).

### Modulation of O-R priming by changes in outcome value

These aforementioned studies did not demonstrate that O-R priming is immediately sensitive to *changes* in outcome value. It is possible that instead outcome value affected the learning process and thereby the strength of the O-R associations. In order to investigate whether behaviour is based on the current desirability of the anticipated outcome, animal researchers have developed the classic outcome-devaluation paradigm. Following an instrumental R-O learning phase, one of the outcomes is devalued (e.g. through satiation) and behaviour is then assessed in extinction. If the subject selectively reduces responding for the now devalued outcome, then it is behaving in a goal-directed manner. With this paradigm, it has been shown that under certain circumstances humans and other animals are able to modify their behaviour based on the currently anticipated positive or negative consequences of their actions (Adams & Dickinson, 1981; Balleine & O’Doherty, 2010; S. de Wit & Dickinson, 2009). However, the critical question here is whether the O-R mechanism gives rise to behaviour that is immediately modulated by outcome value.

To investigate this issue, reinstatement and PIT studies in animals have investigated the effect of outcome devaluation on O-R priming. Against the notion of adaptive motivational modulation of the O-R mechanism, several animal studies have shown that after devaluation of the food outcome through satiation or food aversion (induced sickness), animals will continue to respond for food rewards when primed with a small piece of that food outcome (Eiserer, 1978; Ostlund & Balleine, 2007) or when indirectly primed by Pavlovian cues previously associated with that food outcome (Holland, 2004; Rescorla, 1994). Studies in humans have employed outcome devaluation through, for example, satiation to test whether O-R priming is immediately sensitive to shifts in motivation. Some of these studies, using food and cigarette rewards, report that O-R priming is not reduced when outcomes are no longer desirable (Hogarth, 2012; Hogarth & Chase, 2011; van Steenbergen, Watson, Wiers, Hommel, & de Wit, 2017; Verhoeven, Watson, & de Wit, 2018; Watson et al., 2014). Watson et al. (2014), for example, first trained participants to make one keyboard response for chocolate Smarties and another response for popcorn. In a separate Pavlovian training phase, participants then learned the relationships between abstract patterns and the delivery of these same food outcomes. During a devaluation phase, participants ate one of the foods to satiety. This selective-satiety manipulation was successful as indicated by the fact that participants selectively reduced responding for the devalued reward when tested in the absence of the Pavlovian cues. However, when the patterns associated with either popcorn or Smarties were presented on-screen, participants responded more frequently for the signalled reward, regardless of whether the outcome was currently desired or not. Similarly, Hogarth and colleagues investigated the role of satiation, health warnings, and nicotine replacement therapy but did not find a reduced O-R priming effect for cigarettes in smokers (Hogarth, 2012; Hogarth & Chase, 2011). Together, this series of studies suggests that in the absence of external cues, individuals rely on both the knowledge of instrumental R-O relationships and the motivational significance of those outcomes to behave in a goal-directed manner and choose the still-valuable outcome (e.g. the nonsated food). When triggered by external cues (either directly by outcomes through O-R or indirectly by Pavlovian stimuli through S-O-R), however, the response-priming effect is not flexibly modulated by changes in outcome value. Similar conclusions were reported by Garofalo and Robbins (2017) using an aversive PIT paradigm where the outcomes were aversive sounds presented to participants over headphones. Here, participants continued to make the avoidance responses in the presence of Pavlovian stimuli that signalled the aversive outcomes, even when the headphones had been removed and the sounds could no longer be delivered (i.e. outcome devaluation).

### Factors influencing sensitivity of O-R priming to motivation

The studies reviewed above demonstrate mixed results as to whether O-R priming is sensitive to the motivational value of the outcome. Some of these different findings could be due to when precisely the motivational manipulation took place. In the study of Watson et al. (2014), both outcomes were equally desirable during the R-O training phase before subsequent devaluation of one of them immediately prior to the test phase (see also Garofalo & Robbins, 2017; Hogarth, 2012; Hogarth & Chase, 2011; van Steenbergen et al., 2017). The studies, highlighted above, that observed stronger response priming for high-value outcomes (Muhle-Karbe & Krebs, 2012; Watson et al., 2016), in contrast, tended to use outcomes that already differed in motivational significance at the start of the experiment. It is therefore possible (as suggested for instance by Muhle-Karbe & Krebs, 2012) that stronger associative bonds between response and outcome representations were formed for high-value outcomes during training, leading to differences in the strength of O-R priming at test. Therefore, it is feasible that O-R *learning* is sensitive to outcome value, but that O-R *priming* in the presence of external cues is generally not flexibly modulated by changes in outcome value. This hypothesis does, however, warrant future investigation, as Verhoeven and colleagues did not find any evidence that O-R priming was reduced when participants read health warnings before the training phases compared with a group that read them before the test phase (Verhoeven et al., 2018).

A related issue that should be noted is that not all combined devaluation-PIT studies provided evidence for motivational insensitivity of O-R priming. There have been four human studies that did find that indirect O-R priming was reduced following a posttraining devaluation manipulation (Allman et al., 2010; Eder & Dignath, 2016a, 2016b; Seabrooke, Le Pelley, Hogarth, & Mitchell, 2017). Three of these studies used designs that may have encouraged participants to adopt a more explicit strategy when performing the task—by using a stock market paradigm in which value was instructed (Allman et al., 2010; Eder & Dignath, 2016a) or by presenting novel compound stimuli during the test phase (Seabrooke et al., 2017; see also Claes et al., 2016). Seabrooke et al. (2017), for example, used a modified PIT design where each response was paired with two different food outcomes. During the devaluation phase, taste aversion was used to devalue one of the outcomes associated with each response. Finally during the test phase, participants were presented with a compound stimulus that signalled both one devalued outcome (associated with one response) and one still-valuable outcome (associated with the other response); this novel stimulus may have explicitly signalled to participants that a choice should be made between the two responses. The extent to which participants adopt an explicit strategy as opposed to relying on learned associations is an important variable to consider. Recently, there have been several attempts to show that the PIT effect can, at least in some cases, be driven by explicit, reasoned expectations rather than associative processes. To the degree that PIT is driven by an explicit choice strategy, it could be expected to be sensitive to goal value. It is challenging to ascertain the degree to which associative processes contribute to PIT, but certainly it seems plausible that these can sometimes be overridden. It is likely that, depending on exact task instructions and conditions, participants use different strategies when choosing which outcome to respond for. For example, a unique feature of the O-R priming studies that did show insensitivity to outcome devaluation (Hogarth, 2012; Hogarth & Chase, 2011; Watson et al., 2014) is that participants were instructed during the instrumental (and test) phases that, whilst they would not be told which reward was available, only one reward was available on each trial. Although not formally demonstrated, this instruction likely encourages participants to sample both response keys during the test phase and may therefore make choice behaviour more susceptible to the biasing effect of the cues that are presented. In addition, recent studies have shown that O-R priming can be attenuated, and even reversed, with verbal instructions regarding the informative status of the Pavlovian stimulus (Hogarth et al., 2014; Seabrooke, Hogarth, & Mitchell, 2016). One way to explain these findings is by positing that, in PIT experiments, associative O-R processes can be overridden when an explicit strategy is encouraged. Another source of evidence for a role of explicit reasoning processes in PIT paradigms is observations that the PIT effect only occurs in a subset of ‘aware’ participants who can correctly report the S-O and O-R contingencies (Jeffs & Duka, 2017; Seabrooke et al., 2016). We should, however, point out that these correlational findings do not constitute direct evidence for a causal link between explicit contingency knowledge and behavioural performance.

The other study that provided evidence for reduced outcome-specific PIT after outcome devaluation was conducted by Eder and Dignath (2016b). They used drink outcomes and devalued one of these by adding an aversive-tasting flavour. Although the authors argue that the stronger devaluation treatment (taste aversion) was more effective than other studies that did not find a reduced PIT effect, these results are not in line with animal and human studies that have used similar devaluation methods and still observed intact O-R priming (Holland, 2004; Rescorla, 1994; Seabrooke et al., 2017, Experiment 1). Furthermore, although the outcomes were not presented during the test phase, the devaluation effect was only observed in Experiment 1 when participants experienced the aversive-tasting outcome just prior to, and half way through, the test phase (i.e. the test was arguably not performed in extinction). The devaluation effect was not replicated in Experiment 2 which was performed in extinction. Of course, human behaviour is rarely performed in extinction, and so the study of Eder and Dignath (2016b) does have some ecological validity in that regard, but these results can only offer limited input to the discussion of whether the O-R priming mechanism is *directly* sensitive to changes in outcome value.

In summary, the available evidence suggests that responses associated with high-value outcomes (throughout training and testing) are primed faster and more frequently, lending support to the notion that the O-R priming mechanism is weighted by differences in incentive value of outcomes. However, the fact that some studies found that O-R priming could be demonstrated with aversive outcomes, is surprising (Beckers et al., 2002; Eder et al., 2014). It seems maladaptive for the O-R mechanism to give rise to behaviour that enhances the probability of an aversive outcome, and at first glance is certainly not in line with the idea that this mechanism leads to behaviour that is guided by outcome value. In addition, doubts remain as to whether this mechanism is goal directed in the sense that it is influenced by changes in the current outcome value. Most PIT studies so far have provided evidence for a lack of motivational flexibility, by showing that postlearning reductions of outcome value failed to reduce O-R priming. Finally, it appears that certain paradigms and instructions can cause cue-elicited behaviour to be overridden by explicit strategies, and the contribution of associative processes versus explicit expectations remains a matter of dispute but may prove to be a relevant dimension in future analyses of variability in reward sensitivity of PIT.

## Further points of discussion

### Clinical relevance: Additional route to maladaptive habits

Results from a number of the studies reviewed above suggest that O-R priming can be triggered in a relatively automatic manner, regardless of the motivational significance of outcomes. This has implications for clinical practice as stimuli in the environment can trigger maladaptive reward-seeking responses, as seen, for example, in addiction and obesity (Boutelle & Bouton, 2015; Corbit & Janak, 2016; Hogarth, 2012; Hogarth & Chase, 2011; Watson et al., 2014). Unlike S-R habits which build up over time and are specific to a particular stimulus or context (Balleine & O’Doherty, 2010), O-R priming can generalize to any cue that has previously been associated with the instrumental outcome. Given the insensitivity to outcome devaluation, (S-)O-R priming effects can thus be considered as a highly potent, additional, indirect path to habitual control (in addition to context-bound S-R habitual responding; Watson & de Wit, 2018). Neuroimaging results in humans support this claim as the posterior putamen (involved in habitual S-R behaviour; S. de Wit et al., 2012; Delorme et al., 2016; Liljeholm & O’Doherty, 2012; Tricomi, Balleine, & O’Doherty, 2009) is also implicated during cue-elicited O-R priming (Bray et al., 2008; Prévost et al., 2012; van Steenbergen et al., 2017).

The insensitivity to outcome devaluation displayed by both (S-)O-R priming and S-R habits that are triggered by specific contexts is problematic for many current approaches to treatment that rely on explicitly devaluing outcome value (e.g. by health warnings), as the data reviewed above suggests that this approach will have little effect on reducing cue-elicited responding for signalled rewards (Boutelle & Bouton, 2015; Verhoeven et al., 2018). Indeed, relapse rates remain high in those with drug and alcohol dependence, and weight loss is rarely maintained following dietary interventions (Elfhag & Rössner, 2005; McLellan, Lewis, O’Brien, & Kleber, 2000). This raises the question as to how O-R priming effects could be disrupted or diminished. Attempts have been made to use extinction and relearning procedures to modify the Pavlovian S-O contingencies in order to reduce the ability of stimuli to indirectly trigger O-R behaviour. Reports on the effectiveness of such extinction procedures are, however, mixed. Using a PIT paradigm with various extinction procedures after initial Pavlovian training, Delamater (1996) reported that, in rats, extinction procedures in which the cue was paired with no outcome, or paired with a different outcome, did not reduce the degree to which the cues were still able to elicit anticipation of the original outcome and its associated instrumental response. However, Delamater later reported that if the initial Pavlovian training was brief, then an equivalent number of extinction trials did lead to a reduced PIT effect (Delamater, 2012). In humans, similar manipulations have been used to investigate the effect of S-O extinction on O-R priming (Hogarth et al., 2014; Rosas, Paredes-Olay, García-Gutiérrez, Espinosa, & Abad, 2010). These studies have reported that while the extinction procedure successfully reduced participants’ self-reported expectancy that the outcome would follow the cue, the cue still triggered the instrumental response directed toward the previously associated outcome (Hogarth et al., 2014, Experiment 1; Rosas et al., 2010, Experiments 1 & 2). However, the S-O-R priming effect does show a degree of flexibility as Rosas et al. (2010, Experiment 3) showed that if the Pavlovian stimulus is retrained as a signal that the alternative reward is available, then participants will begin responding for the other reward during the test phase in the presence of that cue. Similarly, Hogarth et al. (2014, Experiment 2) demonstrated that a beer stimulus trained to signal the availability of chocolate caused participants to push more for chocolate. However, through this discriminative extinction training, participants may have learned explicitly that the CS functioned as a hierarchical cue signalling that the instrumental response for the alternative outcome (rather than the signalled outcome) would be reinforced, thereby allowing an explicit strategy to override the associative O-R priming effect.

### Approach and avoidance as instrumental actions

All of the studies that have been considered thus far have examined how presentation or anticipation of an outcome can prime instrumental responses (usually left and right keyboard presses) that previously led to perceptually or affectively similar outcomes. In a related line of research, the focus is on actions that may be inherently valenced—specifically those labelled as ‘approach’ versus ‘avoidance’. A number of studies have systematically investigated how Pavlovian stimuli facilitate and inhibit approach and avoidance actions, revealing a complex interaction between Pavlovian outcome valence, instrumental outcome valence, and action valence (approach or avoid: Geurts, Huys, den Ouden, & Cools, 2013a, 2013b; Huys et al., 2011; Ly, Huys, Stins, Roelofs, & Cools, 2014). In the study by Huys et al. (2011), participants received financial rewards for making both instrumental approach movements (e.g. move the mouse cursor towards a yellow mushroom) and instrumental avoidance actions (e.g. move the cursor away from an orange mushroom). In a Pavlovian training phase, different patterns were associated with financial loss or gain, and these Pavlovian stimuli were then presented as backgrounds while the participants made the instrumental approach and avoidance movements during the test phase. Huys et al. (2011) demonstrated that Pavlovian stimuli associated with winning will only facilitate instrumental approach behaviours, but not instrumental avoid behaviours (even when it concerned a signalled financial outcome of the instrumental avoidance response that was affectively positive; i.e. financial gain). Likewise, Pavlovian stimuli associated with losing money facilitated instrumental avoid behaviours, even when the instrumental avoidance behaviour previously led to winning a financial reward. Similar results have been found using comparable designs (Geurts et al., 2013a; Ly et al., 2014; but see Geurts et al., 2013b, who did not find facilitation/inhibition of specific approach and avoid actions but rather more general effects). Importantly, both the approach and avoidance actions in these aforementioned studies involved ‘going’ (as opposed to ‘not going’), so the results cannot be explained as increased excitation of the motor system following presentation of appetitive Pavlovian stimuli (cf. Chiu et al., 2014). Taken together, these studies provide convincing evidence that the indirect O-R priming effect (in which cue-elicited anticipation of outcomes triggers associated responses) is constrained by additional factors such as action valence.

## Implications for theories of action control

The studies reviewed here highlight that O-R priming can arguably account for a wide variety of behavioural phenomena and is a parsimonious mechanism by which (cue-elicited) outcome anticipation leads to the selection of the appropriate motor patterns required to achieve that outcome. Both the sensory and motivational properties of outcomes can be encoded and mediate the O-R priming effect, and, to some extent, the resulting actions do appear to be weighted by the motivational significance of the anticipated outcomes, in cases where value can impact on the strength of associative learning. However, it appears that O-R priming is not immediately sensitive to (postlearning) changes in the motivational significance of outcomes, as opposed to being dependent on further learning to allow for gradual adjustment of associative weights (in a manner akin to S-R habit reinforcement; Thorndike, 1911). This motivational insensitivity of the O-R mechanism has been demonstrated in outcome devaluation studies. Therefore, it appears that O-R priming is not moderated by immediate motivational factors.

This conclusion appears counterintuitive as there is no logical reason why the O-R priming mechanism should not be modulated by incentive outcome value. In the words of William James, “the fiat, the element of consent, or resolve that the act shall ensue” (James, 1890, p. 501). Ideomotor theorists have proposed that task instructions (“intentional weighting”; Hommel, 2003; Lavender & Hommel, 2007) and/or expected hedonic value (Eder & Rothermund, 2013; Eder et al., 2014) can affect the extent to which a given outcome (or outcome dimension) can activate the associated response. An alternative way in which the O-R pathway could contribute to goal-directed behaviour is if it is supplemented by a general motivational mechanism that simply boosts ongoing motor responses above a certain threshold at times that those outcomes are motivationally relevant (Cartoni et al., 2016). Such a general motivational mechanism has been incorporated in, for example, the revised associative-cybernetic model (S. de Wit & Dickinson, 2009; Dickinson & Balleine, 1994) and has been argued to allow an O-R mechanism—at least under certain circumstances—to yield goal-directed behaviour (S. de Wit & Dickinson, 2016).

The critical question remains, then, as to why most outcome-specific Pavlovian-to-instrumental transfer studies have so far failed to provide evidence for goal-directed behaviour. One explanation is that O-R associations are acquired as a consequence of (stimulus-induced) outcome anticipation (“O”) preceding the reinforced response during training. As a consequence, feed-forward “O”→R associative links can develop that are akin to stimulus-response links. Via these links, the retrieved outcome representation could prime the associated response independently of its current motivational value. Blocked training (as is common in many PIT studies) could also give rise to direct O→R links between the outcome of one trial and the response on the subsequent trial (Ostlund & Balleine, 2007). However, one human PIT study used a concurrent training schedule where the order of trials during instrumental training was randomly intermixed and still reported insensitivity to devaluation (Watson et al., 2014). Another possibility may be that O-R priming is in fact sensitive to outcome value but that the experimental paradigms in use are simply not optimally suited to reveal this. Seabrooke et al. (2017) argue that the standard PIT paradigm is highly sensitive to O-R priming effects for the devalued outcome (as measured in reference to a baseline condition, where participants tend to respond rarely for the devalued outcome). By contrast, there is limited scope for identifying a PIT effect for the valuable outcomes (due to ceiling effects from high levels of responding already present during the baseline trials). Using a modified PIT design, this issue was investigated by Rescorla (1994, Experiment 3) by pairing each Pavlovian stimulus and instrumental response with two rewards: one to-still-be-valuable and one to-be-devalued outcome during test. This way, there was no baseline difference in the two instrumental responses (Pavlovian training: S1-O1 or S1-O2; S2-O3 or S2-O4; instrumental training: R1-O1 or R1-O3; R2-O2 or R2-O4; test phase: O1 and O4 devalued). Nonetheless, the animals performed R1 as frequently in the presence of S1 (with which it shared a devalued outcome) as S2 (sharing a valuable outcome), demonstrating again the insensitivity of OS PIT to outcome devaluation (Rescorla, 1994). Future studies should investigate whether this effect can be replicated in humans (Seabrooke et al., 2017). A final possibility is that O-R priming may simply be an inflexible mechanism that is based purely on learned associations between responses and sensory/affective properties of outcomes that is not at some stage integrated with motivational processes that allow for adjustments on the basis of changes in outcome value. It merely serves, then, to bring to mind available actions, without allowing some of these actions to be prioritised above others in light of current needs and desires. The current paradigms may isolate the sensory O-R priming mechanism and thereby prevent the integration with mechanisms that allow for modulation of behaviour on the basis of outcome value to become visible. If we consider the classic PIT paradigm, this offers a highly impoverished context, in the sense that on each trial only a single outcome is signalled to be available and participants are encouraged to choose between two response alternatives (where not responding is generally not an option). This situation may not be optimally conducive to the engagement of motivational processes, compared, for example, to the general PIT paradigm, where there are more degrees of freedom with the critical variable being the vigour of responding. Therefore, to further assess the validity of models that include the integration of the specific O-R priming effect with a more general motivational mechanism, future studies should adopt more ecologically valid paradigms with multiple cues, responses, and outcomes. As mentioned before, another relevant future direction is to disentangle whether particular task paradigms and instructions engender more explicit strategies in human participants.

Future research along the lines proposed here is needed to determine whether O-R priming can fully account for intentional human behaviour and detail the conditions under which the O-R mechanism is constrained by motivational factors. The explosion of research in this field in recent years means that we will doubtlessly gain further insight into this important fundamental issue. This research should reveal why, in the classic PIT paradigm, O-R priming is inflexible and difficult to adjust or disrupt. This work has important implications not only for theoretical models but also for the appropriate clinical approach towards maladaptive and compulsive behaviours.

## References

[CR1] Aarts H, Dijksterhuis A (2000). Habits as knowledge structures: Automaticity in goal-directed behaviour. Journal of Personality and Social Psychology.

[CR2] Aarts H, Dijksterhuis AP (2000). The automatic activation of goal-directed behaviour: The case of travel habit. Journal of Environmental Psychology.

[CR3] Adams CD, Dickinson A (1981). Instrumental responding following reinforcer devaluation. The Quarterly Journal of Experimental Psychology Section B.

[CR4] Alarcón D, Bonardi C (2016). The effect of conditioned inhibition on the specific Pavlovian-instrumental transfer effect. Journal of Experimental Psychology: Animal Learning and Cognition.

[CR5] Alarcón D, Bonardi C, Delamater AR (2017). Associative mechanisms involved in specific Pavlovian-to-instrumental transfer (PIT) in human learning tasks. Quarterly Journal of Experimental Psychology.

[CR6] Allman MJ, DeLeon IG, Cataldo MF, Holland PC, Johnson AW (2010). Learning processes affecting human decision making: An assessment of reinforcer-selective Pavlovian-to-instrumental transfer following reinforcer devaluation. Journal of Experimental Psychology: Animal Behavior Processes.

[CR7] Asratyan EA (1974). Conditional reflex theory and motivational behaviour. Acta Neurobiologiae Experimentalis.

[CR8] Balleine BW, O’Doherty JP (2010). Human and rodent homologies in action control: Corticostriatal determinants of goal-directed and habitual action. Neuropsychopharmacology.

[CR9] Band GPH, van Steenbergen H, Ridderinkhof KR, Falkenstein M, Hommel B (2009). Action-effect negativity: Irrelevant action effects are monitored like relevant feedback. Biological Psychology.

[CR10] Beckers T, De Houwer J, Eelen P (2002). Automatic integration of non-perceptual action effect features: The case of the associative affective Simon effect. Psychological Research.

[CR11] Boutelle KN, Bouton ME (2015). Implications of learning theory for developing programs to decrease overeating. Appetite.

[CR12] Bray S, Rangel A, Shimojo S, Balleine BW, O’Doherty JP (2008). The Neural mechanisms underlying the influence of Pavlovian cues on human decision making. The Journal of Neuroscience.

[CR13] Campese, V., McCue, M., Lázaro-Muñoz, G., LeDoux, J. E., & Cain, C. K. (2013). Development of an aversive Pavlovian-to-instrumental transfer task in rat. *Frontiers in Behavioral Neuroscience*, *7*. 10.3389/fnbeh.2013.00176 10.3389/fnbeh.2013.00176 PMC3840425 24324417

[CR14] Cartoni Emilio, Balleine Bernard, Baldassarre Gianluca (2016). Appetitive Pavlovian-instrumental Transfer: A review. Neuroscience & Biobehavioral Reviews.

[CR15] Chiu Y-C, Cools R, Aron AR (2014). Opposing effects of appetitive and aversive cues on go/no-go behaviour and motor excitability. Journal of Cognitive Neuroscience.

[CR16] Claes N, Crombez G, Franssen M, Vlaeyen JWS (2016). The impact of Pavlovian cues on pain avoidance: A behavioral study. Learning and Motivation.

[CR17] Claes N, Vlaeyen JWS, Crombez G (2016). Pain in context: Cues predicting a reward decrease fear of movement related pain and avoidance behavior. Behaviour Research and Therapy.

[CR18] Colagiuri B, Lovibond PF (2015). How food cues scan enhance and inhibit motivation to obtain and consume food. Appetite.

[CR19] Corbit LH, Balleine BW (2005). Double dissociation of basolateral and central amygdala lesions on the general and outcome-specific forms of Pavlovian-instrumental transfer. The Journal of Neuroscience: The Official Journal of the Society for Neuroscience.

[CR20] Corbit LH, Janak PH (2016). Habitual alcohol seeking: Neural bases and possible relations to alcohol use disorders. Alcoholism, Clinical and Experimental Research.

[CR21] Corbit LH, Janak PH, Balleine BW (2007). General and outcome-specific forms of Pavlovian-instrumental transfer: The effect of shifts in motivational state and inactivation of the ventral tegmental area. European Journal of Neuroscience.

[CR22] de Wit H (1996). Priming effects with drugs and other reinforcers. Experimental and Clinical Psychopharmacology.

[CR23] de Wit S, Corlett PR, Aitken MR, Dickinson A, Fletcher PC (2009). Differential engagement of the ventromedial prefrontal cortex by goal-directed and habitual behaviour toward food pictures in humans. The Journal of Neuroscience: The Official Journal of the Society for Neuroscience.

[CR24] de Wit S, Dickinson A (2009). Associative theories of goal-directed behaviour: A case for animal–human translational models. Psychological Research.

[CR25] de Wit S, Dickinson A, Braver TS (2016). Ideomotor mechanisms of goal-directed behaviour. *Motivation and cognitive control*.

[CR26] de Wit S, Niry D, Wariyar R, Aitken MRF, Dickinson A (2007). Stimulus-outcome interactions during instrumental discrimination learning by rats and humans. *Journal of Experimental Psychology*. Animal Behavior Processes.

[CR27] de Wit S, van de Vijver I, Ridderinkhof KR (2014). Impaired acquisition of goal-directed action in healthy aging. Cognitive, Affective, & Behavioral Neuroscience.

[CR28] de Wit S, Watson P, Harsay HA, Cohen MX, van de Vijver I, Ridderinkhof KR (2012). Corticostriatal connectivity underlies individual differences in the balance between habitual and goal-directed action control. The Journal of Neuroscience.

[CR29] Delamater AR (1996). Effects of several extinction treatments upon the integrity of Pavlovian stimulus–outcome associations. Animal Learning & Behavior.

[CR30] Delamater AR (2012). Issues in the extinction of specific stimulus-outcome associations in Pavlovian conditioning. Behavioural Processes.

[CR31] Delorme C, Salvador A, Valabrègue R, Roze E, Palminteri S, Vidailhet M (2016). Enhanced habit formation in Gilles de la Tourette syndrome. Brain: A Journal of Neurology.

[CR32] Desantis A, Roussel C, Waszak F (2014). The temporal dynamics of the perceptual consequences of action-effect prediction. Cognition.

[CR33] Dickinson A, Balleine BW (1994). Motivational control of goal-directed action. Learning & Behavior.

[CR34] Dignath D, Pfister R, Eder AB, Kiesel A, Kunde W (2014). Something in the way she moves—Movement trajectories reveal dynamics of self-control. Psychonomic Bulletin & Review.

[CR35] Eder AB, Dignath D (2016). Asymmetrical effects of posttraining outcome revaluation on outcome-selective Pavlovian-to-instrumental transfer of control in human adults. Learning and Motivation.

[CR36] Eder AB, Dignath D (2016). Cue-elicited food seeking is eliminated with aversive outcomes following outcome devaluation. Quarterly Journal of Experimental Psychology (2006).

[CR37] Eder AB, Rothermund K, Mohiyeddini C, Eysenck M, Bauer S (2013). Emotional action: An ideomotor model. *Handbook of psychology of emotions* Vol. 1, Chapter 2.

[CR38] Eder Andreas B., Rothermund Klaus, De Houwer Jan, Hommel Bernhard (2014). Directive and incentive functions of affective action consequences: an ideomotor approach. Psychological Research.

[CR39] Eiserer L (1978). Effects of food primes on the operant behaviour of nondeprived rats. Learning & Behavior.

[CR40] Elfhag K, Rössner S (2005). Who succeeds in maintaining weight loss? A conceptual review of factors associated with weight loss maintenance and weight regain. Obesity Reviews: An Official Journal of the International Association for the Study of Obesity.

[CR41] Elsner B, Hommel B (2001). Effect anticipation and action control. Journal of Experimental Psychology: Human Perception and Performance.

[CR42] Elsner B, Hommel B (2004). Contiguity and contingency in action-effect learning. Psychological Research.

[CR43] Fedorchak PM, Bolles RC (1986). Differential outcome effect using a biologically neutral outcome difference. Journal of Experimental Psychology: Animal Behavior Processes.

[CR44] Garbusow Maria, Schad Daniel J., Sebold Miriam, Friedel Eva, Bernhardt Nadine, Koch Stefan P., Steinacher Bruno, Kathmann Norbert, Geurts Dirk E. M., Sommer Christian, Müller Dirk K., Nebe Stephan, Paul Sören, Wittchen Hans-Ulrich, Zimmermann Ulrich S., Walter Henrik, Smolka Michael N., Sterzer Philipp, Rapp Michael A., Huys Quentin J. M., Schlagenhauf Florian, Heinz Andreas (2015). Pavlovian-to-instrumental transfer effects in the nucleus accumbens relate to relapse in alcohol dependence. Addiction Biology.

[CR45] Garofalo, S., & di Pellegrino, G. (2015). Individual differences in the influence of task-irrelevant Pavlovian cues on human behaviour. *Frontiers in Behavioral Neuroscience*, *9*. 10.3389/fnbeh.2015.00163 10.3389/fnbeh.2015.00163 PMC4478391 26157371

[CR46] Garofalo, S., & Robbins, T. W. (2017). Triggering avoidance: Dissociable influences of aversive Pavlovian conditioned stimuli on human instrumental behavior. *Frontiers in Behavioral Neuroscience*, *11*. 10.3389/fnbeh.2017.00063 10.3389/fnbeh.2017.00063 PMC5388761 28446868

[CR47] Gaschler R, Nattkemper D (2012). Instructed task demands and utilization of action effect anticipation. Frontiers in Psychology.

[CR48] Geurts DEM, Huys QJM, den Ouden HEM, Cools R (2013). Aversive Pavlovian control of instrumental behaviour in humans. Journal of Cognitive Neuroscience.

[CR49] Geurts DEM, Huys QJM, den Ouden HEM, Cools R (2013). Serotonin and aversive Pavlovian control of instrumental behaviour in humans. The Journal of Neuroscience: The Official Journal of the Society for Neuroscience.

[CR50] Gilroy KE, Everett EM, Delamater AR (2014). Response-outcome versus outcome-response associations in Pavlovian-to-instrumental transfer: Effects of instrumental training context. International Journal of Comparative Psychology.

[CR51] Gormezano I, Tait RW (1976). The Pavlovian analysis of instrumental conditioning. The Pavlovian Journal of Biological Science.

[CR52] Gozli DG, Huffman G, Pratt J (2016). Acting and anticipating: Impact of outcome-compatible distractor depends on response selection efficiency. Journal of Experimental Psychology: Human Perception and Performance.

[CR53] Guitart-Masip M, Fuentemilla L, Bach DR, Huys QJM, Dayan P, Dolan RJ, Duzel E (2011). Action dominates valence in anticipatory representations in the human striatum and dopaminergic midbrain. The Journal of Neuroscience: The Official Journal of the Society for Neuroscience.

[CR54] Herwig A, Horstmann G (2011). Action-effect associations revealed by eye movements. Psychonomic Bulletin & Review.

[CR55] Herwig A, Prinz W, Waszak F (2007). Two modes of sensorimotor integration in intention-based and stimulus-based actions. Quarterly Journal of Experimental Psychology (2006).

[CR56] Herwig A, Waszak F (2009). Intention and attention in ideomotor learning. Quarterly Journal of Experimental Psychology (2006).

[CR57] Hogarth Lee (2012). Goal-directed and transfer-cue-elicited drug-seeking are dissociated by pharmacotherapy: Evidence for independent additive controllers. Journal of Experimental Psychology: Animal Behavior Processes.

[CR58] Hogarth L, Chase HW (2011). Parallel goal-directed and habitual control of human drug-seeking: Implications for dependence vulnerability. Journal of Experimental Psychology: Animal Behavior Processes.

[CR59] Hogarth L, Dickinson A, Wright A, Kouvaraki M, Duka T (2007). The role of drug expectancy in the control of human drug seeking. Journal of Experimental Psychology: Animal Behavior Processes.

[CR60] Hogarth L, Retzler C, Munafò MR, Tran DMD, Troisi JR, Rose AK (2014). Extinction of cue-evoked drug-seeking relies on degrading hierarchical instrumental expectancies. Behaviour Research and Therapy.

[CR61] Holland PC (2004). Relations between Pavlovian-instrumental transfer and reinforcer devaluation. Journal of Experimental Psychology: Animal Behavior Processes.

[CR62] Holmes NM, Marchand AR, Coutureau E (2010). Pavlovian to instrumental transfer: A neurobehavioural perspective. Neuroscience and Biobehavioral Reviews.

[CR63] Hommel B (2003). Planning and representing intentional action. The Scientific World Journal.

[CR64] Hommel B (2009). Action control according to TEC (theory of event coding). Psychological Research.

[CR65] Hommel Bernhard, Lippelt Dominique P., Gurbuz Ermine, Pfister Roland (2016). Contributions of expected sensory and affective action effects to action selection and performance: Evidence from forced- and free-choice tasks. Psychonomic Bulletin & Review.

[CR66] Hommel B, Müsseler J, Aschersleben G, Prinz W (2001). The theory of event coding (TEC): A framework for perception and action planning. The Behavioral and Brain Sciences.

[CR67] Huys QJM, Cools R, Gölzer M, Friedel E, Heinz A, Dolan RJ, Dayan P (2011). Disentangling the roles of approach, activation and valence in instrumental and Pavlovian responding. PLOS Computational Biology.

[CR68] James W (1890). *The principles of psychology*.

[CR69] Jeffs S, Duka T (2017). Predictive but not emotional value of Pavlovian stimuli leads to Pavlovian-to-instrumental transfer. Behavioural Brain Research.

[CR70] Konorski J (1967). *Integrative activity of the brain*.

[CR71] Kühn S, Brass M (2010). Planning not to do something: Does intending not to do something activate associated sensory consequences?. Cognitive, Affective & Behavioral Neuroscience.

[CR72] Kühn S, Keizer AW, Rombouts SARB, Hommel B (2010). The functional and neural mechanism of action preparation: Roles of EBA and FFA in voluntary action control. Journal of Cognitive Neuroscience.

[CR73] Kunde W (2004). Response priming by supraliminal and subliminal action effects. Psychological Research.

[CR74] Laurent V, Balleine BW (2015). Factual and counterfactual action-outcome mappings control choice between goal-directed actions in rats. Current Biology: CB.

[CR75] Lavender T, Hommel B (2007). Affect and action: Towards an event-coding account. Cognition & Emotion.

[CR76] Lewis AH, Niznikiewicz MA, Delamater AR, Delgado MR (2013). Avoidance-based human Pavlovian-to-instrumental transfer. The European Journal of Neuroscience.

[CR77] Liljeholm M, O’Doherty JP (2012). Contributions of the striatum to learning, motivation, and performance: An associative account. Trends in Cognitive Sciences.

[CR78] Lotze RH (1852). *Medicinische Psychologie oder die Physiologie der Seele* [Medicinal psychology or the physiology of the soul].

[CR79] Lovibond PF, Colagiuri B (2013). Facilitation of voluntary goal-directed action by reward cues. Psychological Science.

[CR80] Ly V, Huys QJM, Stins JF, Roelofs K, Cools R (2014). Individual differences in bodily freezing predict emotional biases in decision making. Frontiers in Behavioral Neuroscience.

[CR81] Martinovic J, Jones A, Christiansen P, Rose AK, Hogarth L, Field M (2014). Electrophysiological responses to alcohol cues are not associated with Pavlovian-to-instrumental transfer in social drinkers. PLOS ONE.

[CR82] McLellan AT, Lewis DC, O’Brien CP, Kleber HD (2000). Drug dependence, a chronic medical illness: Implications for treatment, insurance, and outcomes evaluation. JAMA.

[CR83] Mok LW, Overmier BJ (2007). The differential outcomes effect in normal human adults using a concurrent-task within-subjects design and sensory outcomes. Psychological Record.

[CR84] Moore JW, Obhi SS (2012). Intentional binding and the sense of agency: A review. Consciousness and Cognition.

[CR85] Morris RW, Quail S, Griffiths KR, Green MJ, Balleine BW (2015). Corticostriatal control of goal-directed action is impaired in schizophrenia. Biological Psychiatry.

[CR86] Muhle-Karbe PS, Krebs RM (2012). On the influence of reward on action-effect binding. Frontiers in Psychology.

[CR87] Nadler Natasha, Delgado Mauricio R., Delamater Andrew R. (2011). Pavlovian to instrumental transfer of control in a human learning task. Emotion.

[CR88] Ostlund SB, Balleine BW (2007). Selective reinstatement of instrumental performance depends on the discriminative stimulus properties of the mediating outcome. Learning & Behavior.

[CR89] Paredes-Olay C, Abad MJF, Gámez M, Rosas JM (2002). Transfer of control between causal predictive judgments and instrumental responding. Animal Learning & Behavior.

[CR90] Pavlov IP (1927). *Conditioned reflexes*.

[CR91] Pavlov IP (1932). The reply of a physiologist to psychologists. Psychological Review.

[CR92] Pezzulo G, Baldassarre G, Butz MV, Castelfranchi C, Hoffmann J, Butz MV, Sigaud O, Pezzulo G, Baldassarre G (2007). From actions to goals and vice-versa: Theoretical analysis and models of the ideomotor principle and TOTE. *Anticipatory behavior in adaptive learning systems*.

[CR93] Pfister R, Janczyk M, Wirth R, Dignath D, Kunde W (2014). Thinking with portals: Revisiting kinematic cues to intention. Cognition.

[CR94] Pfister R, Kiesel A, Hoffmann J (2011). Learning at any rate: Action-effect learning for stimulus-based actions. Psychological Research.

[CR95] Pfister R, Kiesel A, Melcher T (2010). Adaptive control of ideomotor effect anticipations. Acta Psychologica.

[CR96] Pfister R, Melcher T, Kiesel A, Dechent P, Gruber O (2014). Neural correlates of ideomotor effect anticipations. Neuroscience.

[CR97] Prévost C, Liljeholm M, Tyszka JM, O’Doherty JP (2012). Neural correlates of specific and general Pavlovian-to-instrumental transfer within human amygdalar subregions: A high-resolution fMRI study. The Journal of Neuroscience: The Official Journal of the Society for Neuroscience.

[CR98] Quail Stephanie L., Laurent Vincent, Balleine Bernard W. (2017). Inhibitory Pavlovian–instrumental transfer in humans. Journal of Experimental Psychology: Animal Learning and Cognition.

[CR99] Quail Stephanie L., Morris Richard W., Balleine Bernard W. (2017). Stress associated changes in Pavlovian-instrumental transfer in humans. Quarterly Journal of Experimental Psychology.

[CR100] Rescorla RA (1992). Response-outcome versus outcome-response associations in instrumental learning. Animal Learning & Behavior.

[CR101] Rescorla RA (1994). Transfer of instrumental control mediated by a devalued outcome. Learning & Behavior.

[CR102] Rescorla RA, Solomon RL (1967). Two-process learning theory: Relationships between Pavlovian conditioning and instrumental learning. Psychological Review.

[CR103] Rosas JM, Paredes-Olay MC, García-Gutiérrez A, Espinosa JJ, Abad MJF (2010). Outcome-specific transfer between predictive and instrumental learning is unaffected by extinction but reversed by counterconditioning in human participants. Learning and Motivation.

[CR104] Seabrooke T, Hogarth L, Mitchell C (2016). The propositional basis of cue-controlled reward seeking. The Quarterly Journal of Experimental Psychology.

[CR105] Seabrooke Tina, Le Pelley Mike E., Hogarth Lee, Mitchell Chris J. (2017). Evidence of a goal-directed process in human Pavlovian-instrumental transfer. Journal of Experimental Psychology: Animal Learning and Cognition.

[CR106] Shin YK, Proctor RW, Capaldi EJ (2010). A review of contemporary ideomotor theory. Psychological Bulletin.

[CR107] Simon JR, Berbaum K (1990). Effect of conflicting cues on information processing: The “Stroop effect” vs. the “Simon effect.”. Acta Psychologica.

[CR108] Simon JR, Rudell AP (1967). Auditory S-R compatibility: The effect of an irrelevant cue on information processing. The Journal of Applied Psychology.

[CR109] Talmi D, Seymour B, Dayan P, Dolan RJ (2008). Human Pavlovian–instrumental transfer. The Journal of Neuroscience.

[CR110] Thorndike EL (1911). *Animal intelligence: Experimental studies*.

[CR111] Trapold MA (1970). Are expectancies based upon different positive reinforcing events discriminably different?. Learning and Motivation.

[CR112] Trapold M, Overmier JB, Black AA, Prokasy WF (1972). The second learning process in instrumental conditioning. *Classical conditioning II: Current research and theory*.

[CR113] Tricomi E, Balleine BW, O’Doherty JP (2009). A specific role for posterior dorsolateral striatum in human habit learning. The European Journal of Neuroscience.

[CR114] Urcuioli PJ (2005). Behavioral and associative effects of differential outcomes in discrimination learning. Learning & Behavior.

[CR115] van Steenbergen H, Watson P, Wiers RW, Hommel B, de Wit S (2017). Dissociable corticostriatal circuits underlie goal-directed vs. cue-elicited habitual food seeking after satiation: Evidence from a multimodal MRI study. The European Journal of Neuroscience.

[CR116] Verhoeven AAC, Watson P, de Wit S (2018). Failing to pay heed to health warnings in a food-associated environment. Appetite.

[CR117] Vincent R, Hsu Y-F, Waszak F (2016). Category-specific features and valence in action-effect prediction: An EEG study. Biological Psychology.

[CR118] Waszak F, Herwig A (2007). Effect anticipation modulates deviance processing in the brain. Brain Research.

[CR119] Watson P, de Wit S (2018). Current limits of experimental research into habits and future directions. Current Opinion in Behavioral Sciences.

[CR120] Watson P, van Steenbergen H, de Wit S, Wiers RW, Hommel B (2015). Limits of ideomotor action-outcome acquisition. Brain Research.

[CR121] Watson P, Wiers RW, Hommel B, de Wit S (2014). Working for food you don’t desire: Cues interfere with goal-directed food-seeking. Appetite.

[CR122] Watson P, Wiers RW, Hommel B, Ridderinkhof KR, de Wit S (2016). An associative account of how the obesogenic environment biases adolescents’ food choices. Appetite.

[CR123] Zwosta K, Ruge H, Wolfensteller U (2013). No anticipation without intention: Response-effect compatibility in effect-based and stimulus-based actions. Acta Psychologica.

[CR124] Zwosta K, Ruge H, Wolfensteller U (2015). Neural mechanisms of goal-directed behaviour: Outcome-based response selection is associated with increased functional coupling of the angular gyrus. Frontiers in Human Neuroscience.

